# A Brisk and Life-Saving Diagnosis of Pericardial Effusion as the Cause for Recurrent Dyspnea 

**DOI:** 10.24908/pocus.v7i1.15162

**Published:** 2022-04-21

**Authors:** Jacob E Sundberg, Ankit Mehta

**Affiliations:** 1 Department of Hospital Medicine, Regions Hospital Saint Paul, MN USA

**Keywords:** dyspnea, ultrasound, pericardial effusion, diagnosis

## Abstract

Point of care ultrasound (POCUS) is a reliable diagnostic tool for the evaluation of a patient with dyspnea. This case provides an example of an acutely dyspneic patient in which standard evaluation failed to elucidate the true etiology of the patient’s dyspnea. The patient was initially diagnosed with pneumonia but returned to the emergency department with acute worsening of his symptoms despite empiric antibiotics leading to the presumption of antibiotic failure. POCUS revealed a large pericardial effusion requiring pericardiocentesis ultimately leading to the accurate diagnosis. This case highlights the importance of POCUS in evaluating patients with shortness of breath.

## Introduction

Acute dyspnea is a common presentation typically requiring a rapid and thorough evaluation 1, 2. Given myriad etiologies of dyspnea, finding the potential cause for presentation poses a challenge as the underlying cause could be life-threatening. Point of care ultrasound (POCUS) has been noted to expedite a precise diagnosis for the etiology of acute dyspnea, especially in uncertain scenarios 3. The American College of Physicians (ACP) developed guidelines for the appropriate use of POCUS in patients with acute dyspnea in emergent settings. The rationale for these guidelines was based on several considerations including increased proportion of correct diagnosis by 32% when used in addition to the standard diagnostic pathway, improved test accuracy (particularly sensitivity) and no known serious harms 4. In the present report, we describe a clinical scenario of patient presenting with acute dyspnea at the heels of acute hospitalization due to community acquired pneumonia and the use of POCUS that completely changed the diagnostic pathway.

## Case

**Figure 1:**
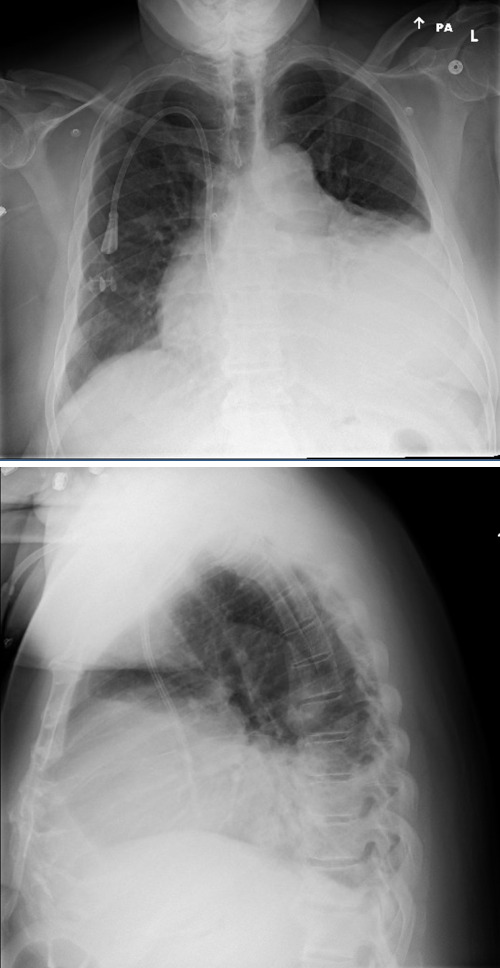
Two view chest x ray showing massive cardiomegaly vs obscuration of left heart border and hemidiaphragm originally diagnosed as left lower lobe infiltrate due to CAP.

**Figure 2:**
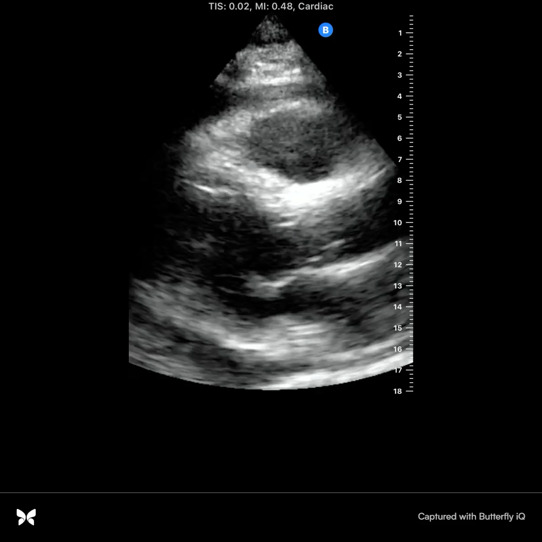
Parasternal long axis view by point of care cardiac ultrasonography revealing moderate to large pericardial effusion.

**Figure 3:**
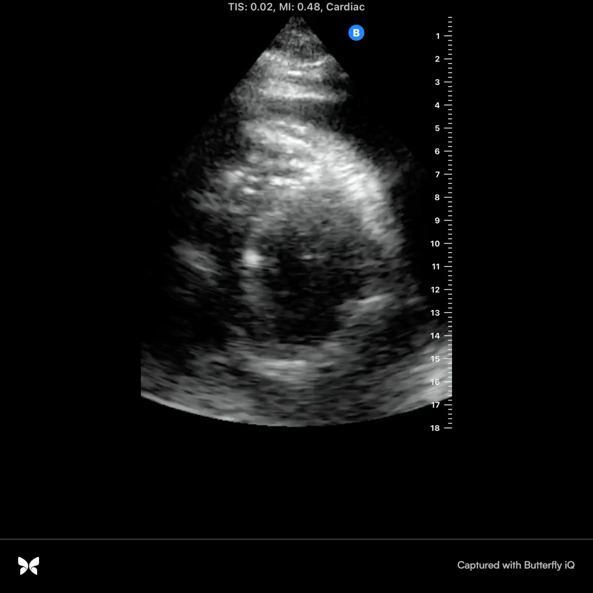
Parasternal short axis view by point of care cardiac ultrasonography revealing moderate to large pericardial effusion.

**Figure 4:**
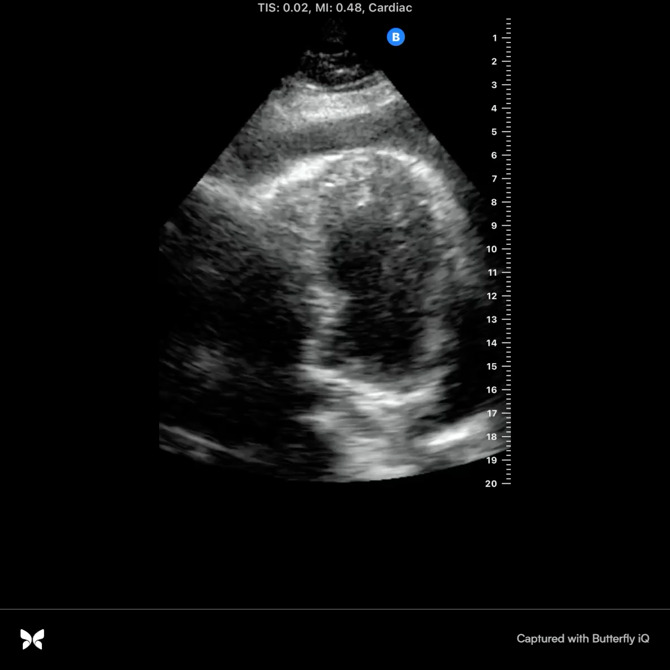
Apical four chamber view by point of care cardiac ultrasonography revealing moderate to large pericardial effusion. This view is foreshortened and unable to completely visualize the atria therefore limiting interpretation.

**Figure 5:**
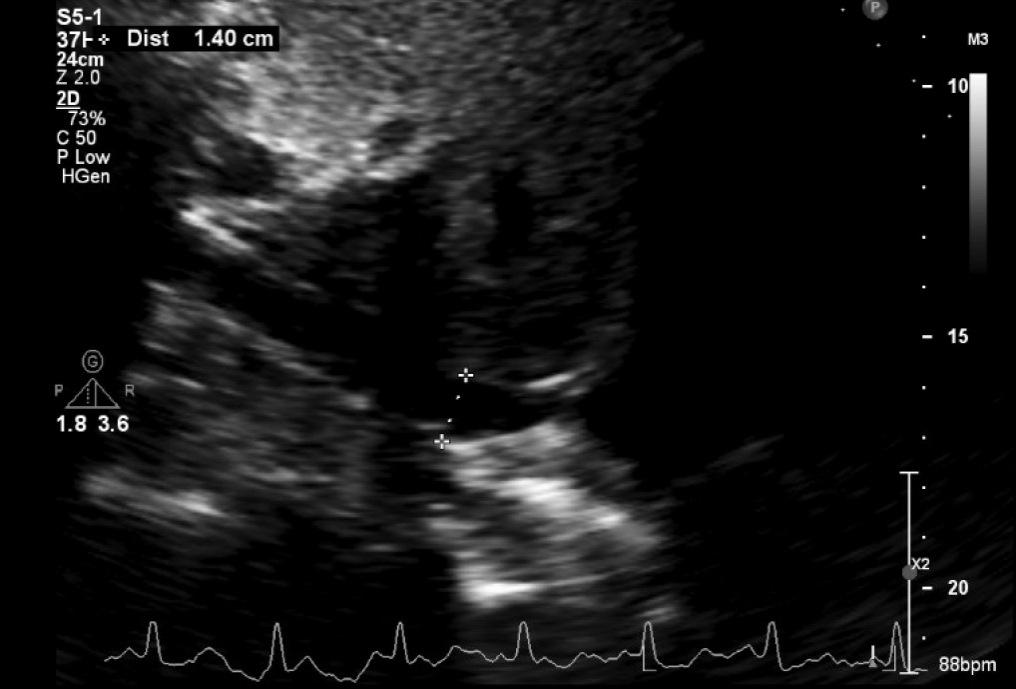
A small caliber IVC with significantrespirophasic variation. This provided reassurance that this was not tamponade physiology.

A 59-year-old man with diabetes mellitus, hypertension, obstructive sleep apnea, dyslipidemia, cerebrovascular disease, and end stage renal disease on hemodialysis presented to the emergency department with progressive dyspnea. He was hospitalized one week earlier and diagnosed with community acquired pneumonia based on his symptoms of exertional dyspnea and chest tightness as well as chest x-ray findings of obscuration of left hemidiaphragm and left heart border and possible infiltrate (Figure 1). He was discharged on empiric oral antibiotics, however his dyspnea continued to progress along with the interval development of dry cough. At baseline he was active, working as a mason, and could easily walk a mile or take a few flights of stairs. Upon return to the emergency department, he was struggling to walk across the room due to dyspnea and exertional chest tightness. He denied nausea, light-headedness, lower extremity swelling, or paroxysmal nocturnal dyspnea. His vital signs were within normal limits. He had no supplemental oxygen needs. He was afebrile with no leukocytosis along with normal troponin and electrocardiogram. He was negative for COVID-19. There was an apparent worsening of left lower lobe infiltrate on chest x-ray. Of note, his last hemodialysis run was shortened to 3 hours due to clotted access. The patient was presumed to have the diagnosis of pneumonia with empiric antibiotic failure. POCUS was performed at the time of admission and revealed moderate to large pericardial effusion (Figures 2-4, online Video S1-3) with redemonstration of consolidation on the left side lung ultrasound with bilateral A profile. His inferior vena cava (IVC) showed considerable respiratory variation (Figure 5). This essentially ruled out tamponade physiology, as IVC plethora is a quite sensitive echocardiographic sign of cardiac tamponade 5, 6.

A STAT echocardiogram and cardiology consultation was ordered which confirmed the findings of the POCUS study. IVC respiratory variation was reassuring in ruling out tamponade physiology and an emergent need for intervention. Therefore, the patient was taken for pericardiocentesis the following morning whereby 870 milliliters of bloody fluid was drained. Pericardial fluid was negative for bacterial and fungal cultures, acid fast bacilli, and cytology for malignancy. His anti-nuclear antibody was noted to be positive (>1:640) along with an elevated C-reactive protein (CRP) and erythrocyte sedimentation rate (ESR). During the hospital course, he unfortunately developed rapid accumulation of the pericardial effusion necessitating a pericardial window. Rheumatology was consulted and further studies including myeloperoxidase and serum proteinase 3 antibodies (MPO/PR3), rheumatoid factor (RF), cyclic citrullinated peptide antibody (anti-CCP), complements (C3/C4), and double stranded DNA antibodies (anti-dsDNA) were sent. These studies were subsequently noted to be negative. The differential diagnosis for this patient’s pericardial effusion remains uremia related vs an autoimmune etiology. His dyspnea improved and the patient was discharged to home with close cardiology and rheumatology follow up. 

## Discussion

The present case highlights the vital role of POCUS in diagnosing a patient accurately with a symptomatic pericardial effusion. POCUS is a diagnostic tool that has increasingly been used for rapid evaluation of the acutely dyspneic patient, especially in emergent settings. It is particularly useful given its wide applicability to assist with the rapid diagnosis and treatment for a patient with shortness of breath 7, 8. Multiple studies have validated its diagnostic accuracy and possible superiority to standard work up performed in these patients 9, 10. A study of patients presenting with dyspnea or chest pain demonstrated that the POCUS exam revealed clinically relevant findings among 79% of patients and led to alteration of the primary diagnosis among 28% of patients. Additionally, time to diagnosis was significantly shorter among patients in the POCUS group compared with the control group with median time of 5 hours vs. 24 hours 3. In this case, POCUS changed the plan of care rapidly and diagnosed a potentially life-threatening acute condition that required early intervention. This further supports the benefits of early and often use of bedside ultrasonography for management of patients presenting with shortness of breath.

## Statement of Ethics

Informed consent was obtained from the patient by the authors. The patient has consented to the use of de-identified images, video clips, and health information to be published within the journal.

## Disclosures

The authors have no conflicts of interest to declare.

## Supplementary Material

 Video S1 Parasternal long axis view by point of care cardiac ultrasonography revealing moderate to large pericardial effusion.

Video S2 Parasternal short axis view by point of care cardiac ultrasonography revealing moderate to large pericardial effusion.

Video S3 Apical four chamber view by point of care cardiac ultrasonography revealing moderate to large pericardial effusion. This view is foreshortened and unable to completely visualize the atria therefore limiting interpretation.
